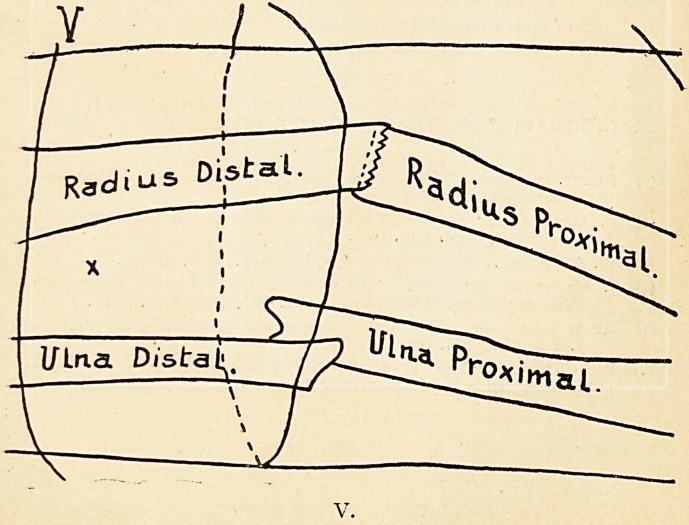# Twin X-Ray Representation and the Reflecting Stereoscope

**Published:** 1905-09

**Authors:** William Cotton


### TWIN X-RAY REPRESENTATION AND THE REFLECTING STEREOSCOPE.

BY

William Cotton, M.D.

---

Owing to the centrifugal nature of its projection, a single X-ray representation of a foreign body or an osseous lesion puts its interpreter into a dilemma when he seeks to correctly pencil the outlines of his radiograph for the information of the practical surgeon. Thus (in Illustration I.) the transparent X-ray silhouettes of a comminuted fracture A may be represented as either B or C, according to the conventions of ordinary graphic representation, namely that objects nearer the station point of the eye eclipse or obscure the more remote. Except in a few special cases of objects whose perspective appearance is otherwise already known, this essential ambiguity is best resolved by the well-known stereoscopic methods of taking and viewing a twin pair of radiographs, or even their outlines—whenever in fact it is necessary for practical purposes visually to determine the relative distance of the invisible objects portrayed

I.

in the directions of vision—as was actually done in the foregoing instance, where B was found to be the more proper transcript of a real injury. In this case the stereoscopic blending of the pair of outline silhouettes (of which A B and C represent one member) appears to demonstrate that points in an object measurably distant from each other, when they fall upon physiologically corresponding parts of the two retinæ, may appear single.[1]

In this Journal for September, 1902, is given an example of a foreign body adapted for viewing in Brewster's refracting stereoscope (in the form provided with an interocular partition and associated in America with the name of Oliver Wendell Holmes); the present paper is intended to exemplify fractured and displaced bones in the reflecting instrument of Wheatstone.[2]

Dr. A. W. Peake, who had charge of the case, has kindly furnished the following particulars:—"W. W., æt. 10¼ years, while at play on June 9th fell from the top of a gate and fractured his left forearm. On examination both bones were found broken at about the middle of the shaft, and the usual signs of shortening with angular displacement were present. About six years ago the boy sustained a similar injury to the same part."

On June 19th the limb (put up in its retentive apparatus in the usual way, and with the elbow bent at a right angle and in the mid-position between pronation and supination) was twice radiographed in succession with the flexor surface towards the sensitive film, on separate plates, by a focus tube which was moved across the long axis of the forearm. A wooden cube, 1 inch in the side, was simultaneously taken (in the same relative position to the forearm as far as possible each time) as a guide to the perspective. It had been thinly coated with subnitrate of bismuth suspended in gum. Two originally vertical pins would serve as a somewhat similar device for finding the

[1] If this experimental observation be confirmed, existing theories as to the nature of binocular vision will need to go into the melting-pot once more.

[2] There remains over for application Rollmann's well-known two-colour method, which promises to lend itself readily to X-ray uses.

points of sight. The successive stations of the focus tube were $2\frac{5}{8}$ inches apart. The anti-cathodal centre of emission was about 14 inches above the plane of the two plates, as ascertained by shadowing the cube *in situ* upon the developed plate by means of a small luminous flame.

The pair of radiographs thus obtained may be reduced in size by ordinary photographic methods, certain precautions being taken to prevent exaggeration and misdirection, and thereafter examined in the familiar refracting stereoscope; or in their original size they may be directly examined in the less familiar reflecting stereoscope (Illustration II.).[1]

II.

Even in a beleaguered city it should not be difficult to construct one of these. A very passable one may be made out of a couple of small rectangular pieces of looking-glass, an empty cigar box, two rubber book-bands, and a few wooden boards and blocks on some firm support. The essential part is in the centre, and consists of two plane mirrors $\epsilon$ and $\varphi$ measuring a few inches each way, meeting at right angles along a vertical line, and facing respectively the observer's two eyes.

[1] There seems to be no particular reason why (in certain circumstances and with certain precautions and adjustments) one should not simultaneously combine the X-ray and the photographic views of the same objects stereoscopically.

This is mounted on a base that can be slid backwards and forwards, right and left. On the right and left of the mirrors are placed moveable supports for the stereoscopic photographs that are to be visually blended. After careful levelling and adjustment of the photographs on their supports (or otherwise mounted on sliding carriers that can be moved to and fro along the cross-piece), equidistant from the mirrors, the observer gazes with one eye into $\epsilon$ and the other into $\varphi$, keeping his forehead near and opposite to the angle between the mirrors, and looking through them, as it were, towards a point about as far beyond them as the photographs are to the side.

On sliding the base of the mirrors backwards and forwards he will, if he keeps his head steady and level, find the separate plane images slipping over each other from side to side and suddenly coalescing (within certain critical limits antero-posteriorly) into a single solid image as it were of a triply extended real object or collection of objects. At night a good candle or lamp may very conveniently be placed on the top of the mirrors, and the simple apparatus described may be elaborated further in the direction of providing trans-illumination of the component radiographs by making their supports hollow to contain suitable lights and frosted or opaline screens, or the lateral supports might be made to rotate and display a succession of photographs.

According as we place the two negatives (or prints) at either end of the cross arm, two prints (or negatives) appear respectively in the mirrors $\epsilon$ and $\varphi$, owing to the reversing action of the plane mirrors. When the respective pairs of plane images blend stereoscopically we get four distinct and recognisable triply-extended or stereoscopic images behind the mirrors, at a distance from the eyes corresponding to the original distances of the objects portrayed from the focus tubes. These results merit tabulation.

Let the two positions of the focus tube be represented in order by the letters **e** and **f**, then **E** and **F** represent the corresponding negatives and **Ǝ** and **F** the corresponding prints :—

### LEFT FOREARM X-RAYED AS DESCRIBED.

*(Illustrations III. and IV.)*

| No. | Radiographs as they appear respectively in mirrors ϵ and φ | | Perspective as tested by appearance of wooden cube dusted with bismuth. | Appearance of Limb. |
|---|---|---|---|---|
| I. | E | F | Correct binocular or stereoscopic perspective. | Left forearm through and from back. |
| II. | F | E | Pseudoscopic conversion of relief. | Right forearm through and from front. |
| III. | ꟻ | Ǝ | Correct binocular or stereoscopic perspective. | Right forearm from back. |
| IV. | Ǝ | ꟻ | Pseudoscopic conversion of relief. | Left forearm from front. |

There are other combinations as we invert the radiographs, &c., but the above comprise all the cases for the erect position of the object, the distal extremity of the limb being at the top of the picture. Also it is assumed that the distance of the photographs from the mirror, together with that from the mirror to the eye, is much the same as that of the focus tube from the photographic plate at the time of taking, but there is very great latitude and longitude permissible.[1]

In my opinion No. I. is the only proper combination, though No. IV. might pass muster in a court of law; Nos. III. and IV. are through the looking-glass creations, and Nos. II. and IV. appear to be of the nature of "visual surds" respectively to Nos. I. and III. There appear to be at least two and thirty ways of arranging the components of an X-ray or ordinary photographic couple, and though only two of them are right, nevertheless they may all give recognisably different stereoscopic images.

[1] It would seem that the somewhat perplexing relationships of these diverse imaginary figures come well within the purview of the science of Quaternions, and perhaps even the nervous connections of the brain itself, in the discharge of its stereoscopic functions in binocular vision.

III.

IV.

The system of interpretation of single and paired X-ray representations set forth by me in this Journal and elsewhere is not that generally accepted, based as this is on the rules of *Perspective*, and the use exclusively of the *X-ray negative viewed from its face* as the only true equivalent of the ordinary photographic print. It is, however, a very fair subject for discussion, the existing text-books (on this matter, at any rate) giving no consistent or definite guidance. For example, according to recognised clinical methods the percussion outline of the heart in a big man is fairly *similar* to and reliably comparable with that in a small man, apart from alteration by disease. But on my principles, unless the distance of the focus tube from the subject is proportional to the thickness of these two individuals, the X-ray shadow outline on the chest of the one man is not truly *similar* to or comparable with that in the other. And, further, on my principles, even when the last condition is attended to, the percussion outline and the X-ray shadow outline of an internal organ do not appear to be ever mathematically similar and directly *inter se* comparable.

V.

I am indebted to Mr. Thomas Clark for the originals of the accompanying radiographs and radiographic outlines, the twin negatives being produced with the aid of an electrical influence machine designed and constructed by himself. The exposures were of two minutes each.

As a practical result of the methods used, the radiographer is able to assure his surgical colleague (without any personal examination of the case) that the distal parts of both bones, especially the ulna, are tending to be drawn past the proximal portions, and posteriorly to the latter, though perhaps a good deal of the apparent overlapping of the fragments of the ulna is due to the exaggeration of a close and short perspective. Graphically, the condition of things might be roughly sketched as in Illustration V. If this be held with one eye opposite the point of sight (marked x) at about a distance equal to that which the sensitive plate originally had from the focus tube, the parts depicted will appear under the same angles as they would have appeared in the left forearm in question (viewed through its back from the place where the focus tube was stationed at the time of taking) if the parts had been correspondingly transparently visible.

---

### BIBLIOGRAPHY OF STEREOSCOPIC X-RAY REPRESENTATION.

(1) Elihu Thomson. "Stereoscopic Röntgen Pictures."—*The Electrician*, March 13th, 1896.

(2) P. Czermak. "Stereoscopic Pictures with the Röntgen Rays"—*Photography*, Sept. 24th, 1896.

(3) W. S. Hedley. "Radiostereoscopy."—*Lancet*, 1898, i. 639.

(4) J. Mackenzie Davidson. "Remarks on the Value of Stereoscopic Photography and Skiagraphy."—*Brit. M. J.*, 1898, ii. 1669.

(5) Hugh Walsham. *Stereoscopic Skiagraphy in Diseases of the Chest.*—J. & A. Churchill, 1899.

(6) W. Cotton. "The True and the False Perspective of X-ray Representation."—*Archives of the Röntgen Rays*, July, 1902.

## Figures and Tables

**I. f1:**
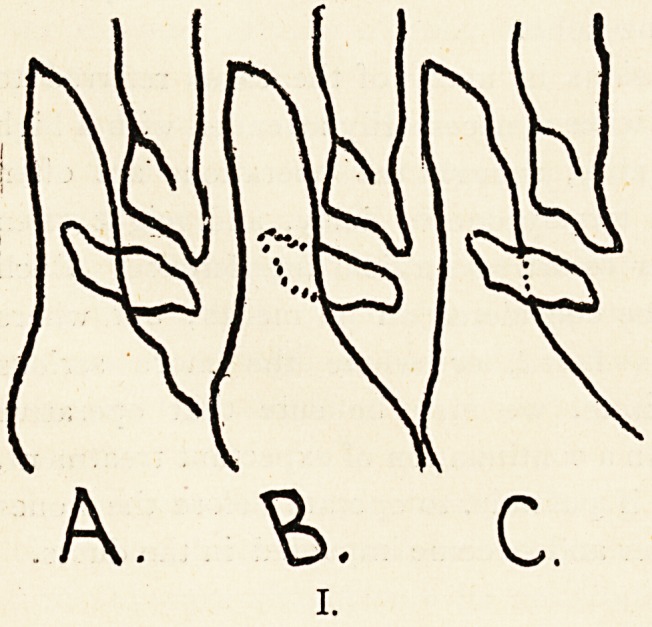


**II. f2:**
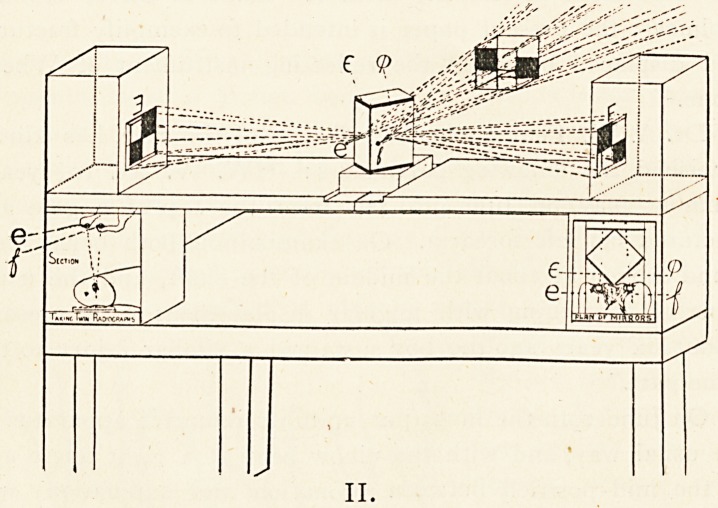


**III. f3:**
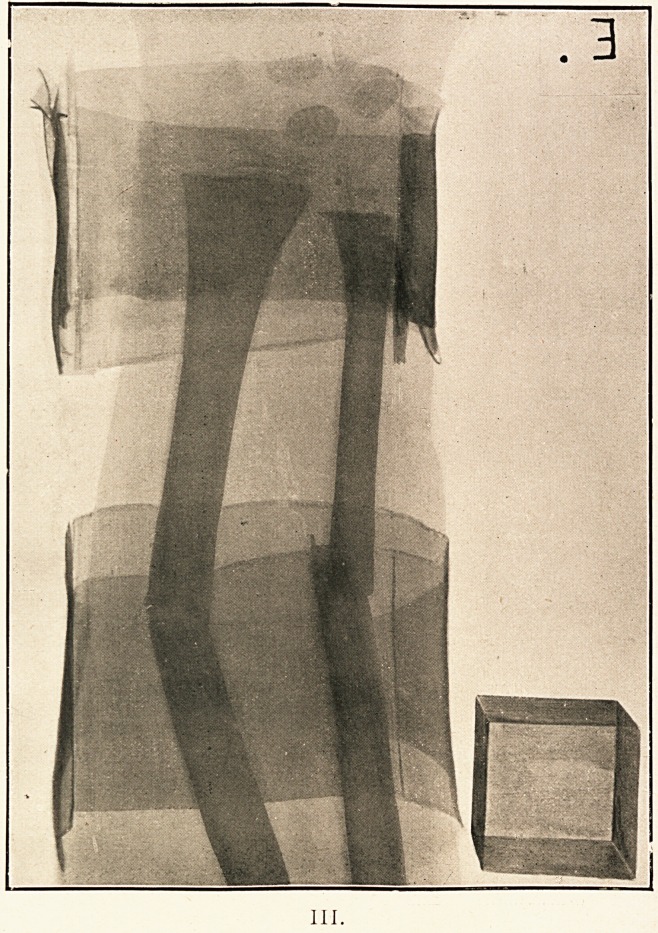


**IV. f4:**
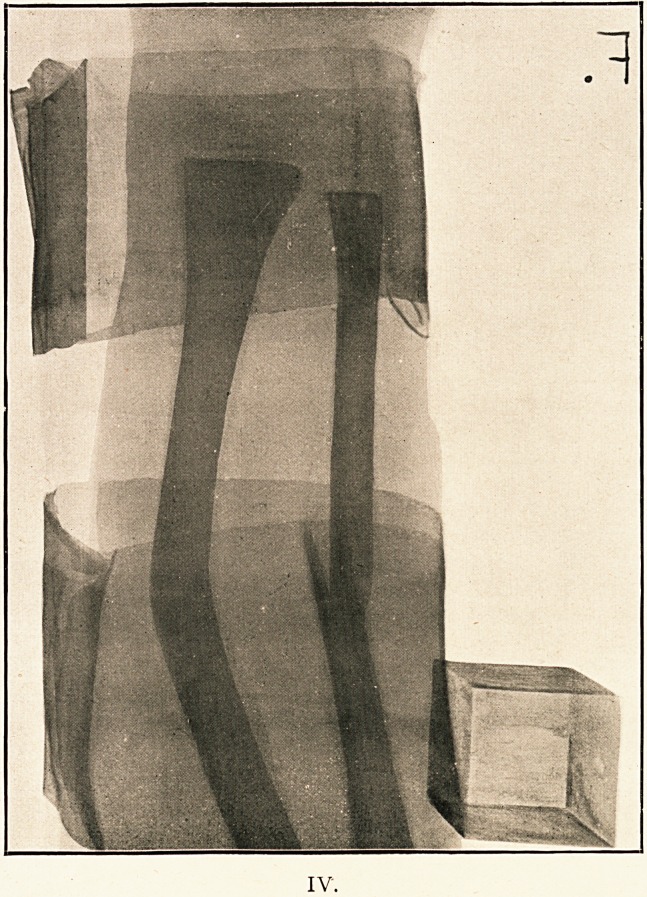


**V. f5:**